# Asymmetric Reflection Induced in Reciprocal Hyperbolic Materials

**DOI:** 10.1021/acsphotonics.2c00551

**Published:** 2022-07-20

**Authors:** Xiaohu Wu, Cameron A. McEleney, Zhangxing Shi, Mario González-Jiménez, Rair Macêdo

**Affiliations:** † Shandong Institute of Advanced Technology, Jinan 250100, Shandong, China; ‡ James Watt School of Engineering, Electronics and Nanoscale Engineering Division, University of Glasgow, Glasgow G12 8QQ, United Kingdom; § School of Chemistry, University of Glasgow, Glasgow G12 8QQ, United Kingdom

**Keywords:** hyperbolic materials, phonons, polarization, reflection, polaritons, reciprocity

## Abstract

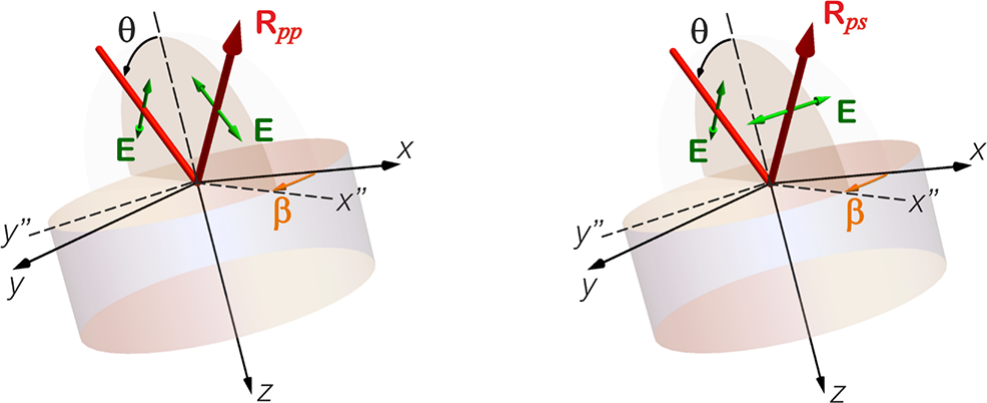

Reflection is one of the most fundamental properties of light propagation. The ability to engineer this property can be a powerful tool when constructing a variety of now ubiquitous optical and electronic devices, including one-way mirrors and antennas. Here, we show from both experimental and theoretical evidence that highly asymmetric reflection can be induced in reciprocal hyperbolic materials. This asymmetry stems from the asymmetric cross-polarization conversion between two linearly polarized waves, an intrinsic and more exotic property of hyperbolic media that is bereft of research. In addition to angle-controllable reflection, our findings suggest that optical devices could utilize the polarization of the incident beam, or even the polarization of the output wave, to engineer functionality; additionally, in hyperbolic slabs or films, the asymmetry can be tailored by controlling the thickness of the material. Such phenomena are key for directional-dependent optical and optoelectronic devices.

## Introduction

Controlling and modulating light propagation has long been a subject of intense interest, as it has enabled several key technological advances through the years.^1−4^ More recently, considerable attention has been paid to the capability to select light propagating in a specific direction.^5−8^ In particular, there has been tremendous interest in the type of propagation through some materials where light traveling forward shows a different behavior compared to when it is traveling backward. This “asymmetric” light propagation is the basis for a variety of optical technological applications; an example of this is signal-processing devices.^9,10^ Nonreciprocal materials, such as magneto-optical materials and Weyl semimetals, have been investigated as a way to achieve asymmetric propagation of light because of their ability to break time-reversal symmetry.^11−15^ For instance, the reflection of light has been successfully modulated using bulk magneto-optical materials,^16−19^ as surface magnon-polaritons are highly nonreciprocal. In antiferromagnets, for instance, the hyperbolic behavior associated with magnon-polaritons has been shown to be an excellent mechanism for engineering reflectivity,^20^ and very recently, nonreciprocal reflection has been shown to be dramatically enhanced in structured magnetic gratings.^21^ However, magneto-optical materials of this sort can be incompatible with compact integrated electronic devices, as they commonly require strong externally applied magnetic fields or low temperatures.^20,22^

As an alternative to magneto-optical materials, studies of synthetic materials and metamaterials are ongoing.^10,23^ With the development of nanofabrication, the study of metasurfaces has become a popular topic;^24^ several of these are now suggested to support asymmetric wave propagation.^25−27^ For instance, asymmetric hybridized metamaterials have been engineered to display polarization-dependent asymmetric transmission.^28^ However, they commonly require sophisticated fabrication processes. Many anisotropic materials have also been proposed as a route to realize asymmetric optical properties.^29−35^ For example, asymmetric wave propagation can be supported in reciprocal hyperbolic materials when the anisotropy axis is “bent”, i.e., neither parallel nor perpendicular to the surface of the crystal.^36^ In such cases, while the optical axis always lies in the plane of incidence, absorption and transmission can still be asymmetric. Reflection, on the other hand, is always symmetric due to the Helmholtz reciprocity principle.^34,35^

Here, we introduce the fundamental principle upon which one can achieve asymmetric reflection in reciprocal bulk materials. This originates from the emergence of asymmetric cross-polarization conversion between two linearly polarized waves, which does not violate the Helmholtz reciprocity principle. We explore not only this feature, but also investigate its origin to identify how it can be optimized in order to increase the asymmetry in the reflective surface. First, we prove our fundamental idea theoretically for a linearly polarized wave incident on a semi-infinite hyperbolic crystal. By tailoring the angle of the plane of incidence with respect to the plane of the anisotropy axis, it is possible to design regions wherein asymmetric reflection of linearly polarized incident light is observed. Second, we experimentally demonstrate the concept using the example material crystal quartz, a prime example of low-loss, natural hyperbolic media. In addition, crystal quartz has several active phonon resonances across the far- to mid-infrared frequency band, making it an exciting candidate to be employed in novel devices such as infrared photodetectors^37,38^ and energy conversion devices,^39^ both of which have been shown to be applications that can benefit from asymmetric optical devices. Finally, we discuss further a combination of these mechanisms that can be used to optimize the asymmetries in the intensity of the reflected beam of linearly polarized incident light bouncing off the surface of a hyperbolic material.

## Results

### Premise for Asymmetric Reflection

**Figure 1:**
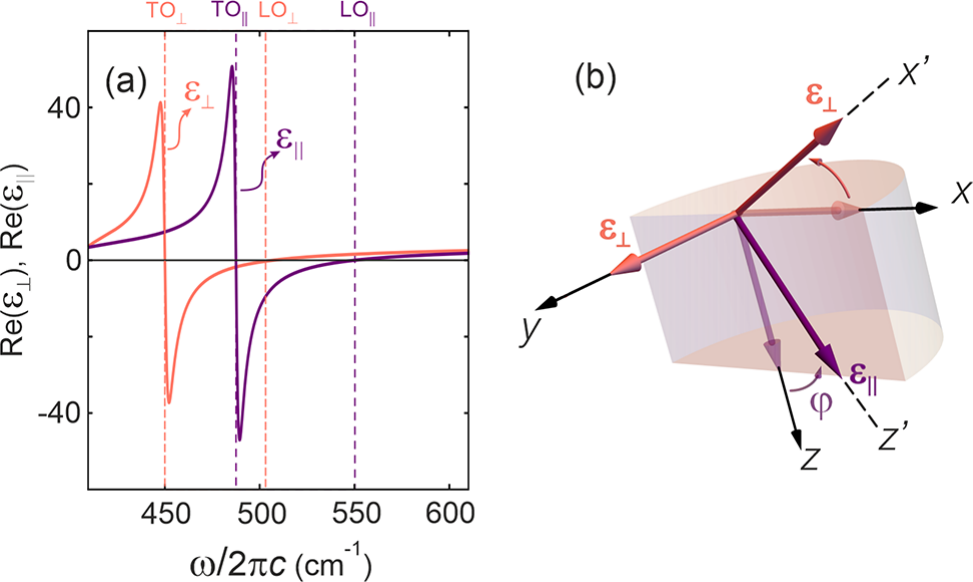
(a) Crystal quartz’s dielectric tensor components parallel and perpendicular to the crystal anisotropy axis, ε_∥_ and ε_⊥_, respectively. (b) Crystal geometry showing the direction of the anisotropy (ε_∥_) axis and plane of rotation (*x*–*z*) used in this work.

Let us start by introducing the example hyperbolic material, which here is chosen to be crystal quartz.^9,29^ This is a naturally occurring hyperbolic material with uniaxial anisotropy and low damping. In this crystal, the condition for hyperbolic dispersion is met in several regions across the infrared spectra due to infrared-active phonon resonances, making one of the principal components of the crystal’s permittivity tensor, , of opposite sign to the other two principal components.^40,41^ These regions can be generally found between the transverse optical (TO) phonon frequencies and the longitudinal optical (LO) phonon frequencies.^42,43^ Here, we will focus on the frequency range between 400 and 600 cm^–1^ (corresponding to free-space wavelengths between 16 and 25 μm) where there exist two regions where the component of  parallel to the anisotropy, ε_∥_, is of an opposing sign to the two components perpendicular to it, ε_⊥_, as shown in Figure 1a.

The geometries in which hyperbolic materials are typically studied are those wherein the anisotropy either lies parallel or perpendicular to the material’s surface.^44−46^ The more common case being that where the material’s surface is perpendicular to the anisotropy axis,^47^ and the less common case being that where the optical axis is parallel to the interface. The latter case, however, has been investigated using materials such as an effective photonic-crystal,^48^ but have gained increased attention with the advent of two-dimensional hyperbolic crystals.^44,49^ Here, we will be concerned with a geometry where the anisotropy axis (represented in the dielectric tensor by the component ε_∥_ and the direction depicted in Figure 1b) is rotated so that it lies neither parallel nor perpendicular to the surface. Instead, it is at angle φ, measured with respect to the *z* axis. While φ can rotate by any amount, in this work we will focus on the case of φ = 45°, where the anisotropy (or the crystallographic *c*-axis) has equal components in both parallel and perpendicular directions to the crystal surface. This mechanism has, in fact, been previously used to show how negative refraction can be obtained in a natural material at optical frequencies.^50^ More recently, this rotation angle in hyperbolic materials has been termed as the “bending angle”, as it can be used to control (or bend) the refracted beam.^34^ It is important to note here that this rotation can be applied to any hyperbolic material, natural or artificial (hyperbolic metamaterials), and so the findings to follow should also be expected to emerge in any hyperbolic system.

**Figure 2:**
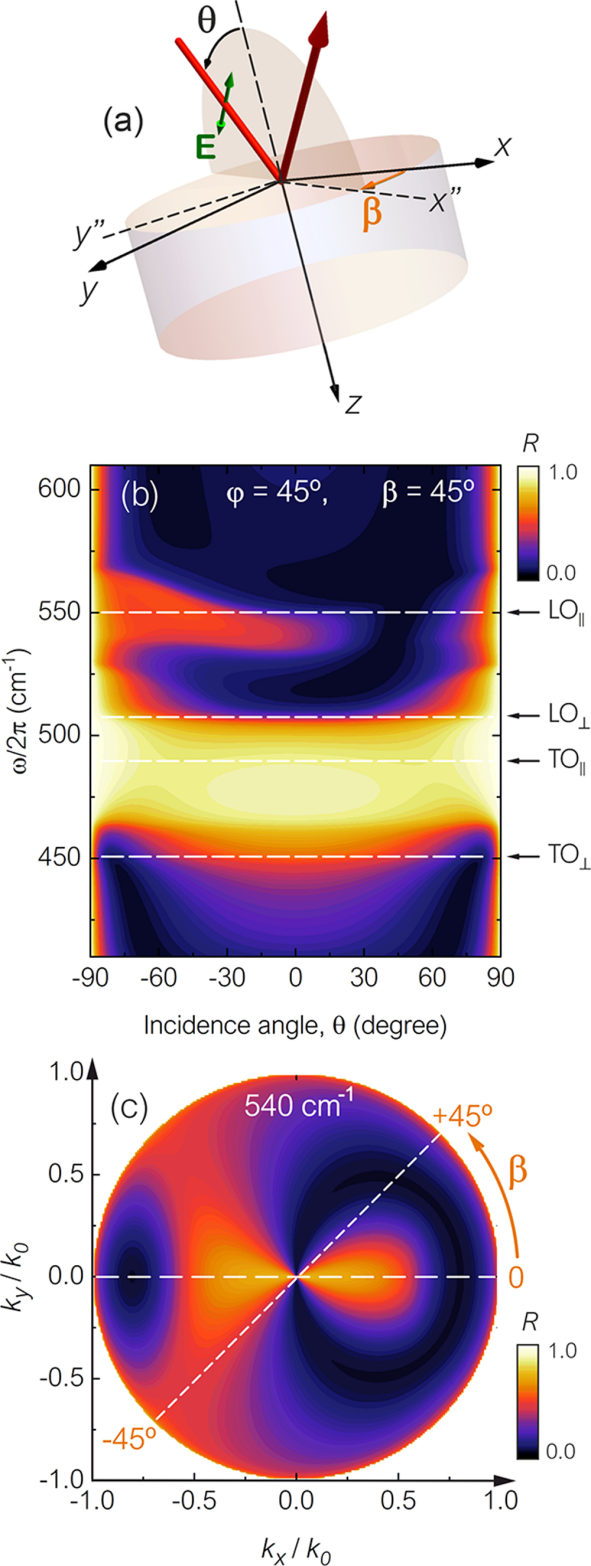
(a) Schematic of the geometry for reflection of a TM-polarized beam incident with an angle θ from air at the surface of a hyperbolic material. Here, the incidence plane is rotated in the *x*–*y* plane by an azimuthal angle β. (b) Reflectance, *R* = rr*, off a semi-infinite quartz crystal as a function of the incident angle θ and the wavenumber ω when both the azimuthal angle, β, and the bending angle φ (see Figure 1b) are 45°. The reflectance as a function of the wavevector in the *k*_*x*_–*k*_*y*_ space (corresponding to a rotation of 360° in β) at 540 cm^–1^ is given for φ = 45° in panel (c).

Let us now investigate the reflection of a transverse magnetic (TM) polarized beam incident at the surface of a semi-infinite hyperbolic material, such as depicted in Figure 2a. Here, in addition to a rotated anisotropy, as that discussed in Figure 1b, the incidence plane is also allowed to rotate around the *z* axis by an azimuthal angle β. Where β is the angle between *x* and its rotated projection *x*″. A rotation of this kind is particularly interesting as in most works investigating the optical properties of hyperbolic media, the incident plane typically lies in the *x*–*z* plane. However, as we will see in what follows, a rotation of the plane of incidence can be used to mediate the emergence of asymmetric reflection in a reciprocal material.

Now, let us look at the reflection for when both the azimuthal angle and the anisotropy angle are set to 45°. This leads to spectral regions of highly asymmetric reflectance, as shown in Figure 2b. Although a rotation of the anisotropy has been demonstrated to generate asymmetric absorption^35^ as well as new regions where maximum reflection is observed,^34^ the reflectivity itself is always symmetric with respect to the incident angle for all frequencies if the incidence plane lies in the same plane as the anisotropy (β = 0). This is in stark contrast to what we see here when the plane of incidence is also rotated, as we can see a strong asymmetry at wavenumbers in the 540–560 cm^–1^ band. For incidence angles around θ = −50°, the reflection is at a maximum, but at θ = +50°, its positive counterpart, it is at a minimum.

In order to further investigate the asymmetric behavior, let us take ω/2π = 540 cm^–1^ as our example frequency and look at the reflection as a function of the wavevector in the *k*_*x*_–*k*_*y*_ space; this corresponds to a 360° rotation of the azimuthal angle, β, and it is shown in Figure 2c for the same bending angle used in Figure 2b of φ = 45°. One can see that the reflection is symmetric on *k*_*y*_ (comparing the upper and lower halves of the circle contour plot) and asymmetric on *k*_*x*_ (comparing the left- and right-hand side of the circle contour plot). The diagonal, short-dashed line shows the equivalent cross-section of Figure 2b, where the top right quadrant gives positive incident angles, and the bottom left quadrant gives negative incident angles. We can confirm from this that while there is low reflection for all negative angles, a region of positive incident angles display near perfect reflection.

This is in stark contrast to the case of β = 0 (long-dashed line in Figure 2c), where the reflection is symmetric.^34^ However, it should be noted that this effect emerges as a combination of, or interplay between, β and φ. In fact, if there is no rotation of the anisotropy, the azimuthal angle does not actually matter. This can be understood by simply looking at the geometry of the system (shown in Figure 1b). First, take the anisotropy (ε_∥_) to lie along *z* and then note that in crystal quartz the permittivity along *x* and *y* takes the same value at all frequencies. It is now easy to see that if we rotate the system around the *z* axis, as shown in Figure 2a, it will make no difference for propagating electromagnetic fields, as the components of  along *x* and *y* are identical (i.e., ε_⊥_).

**Figure 3:**
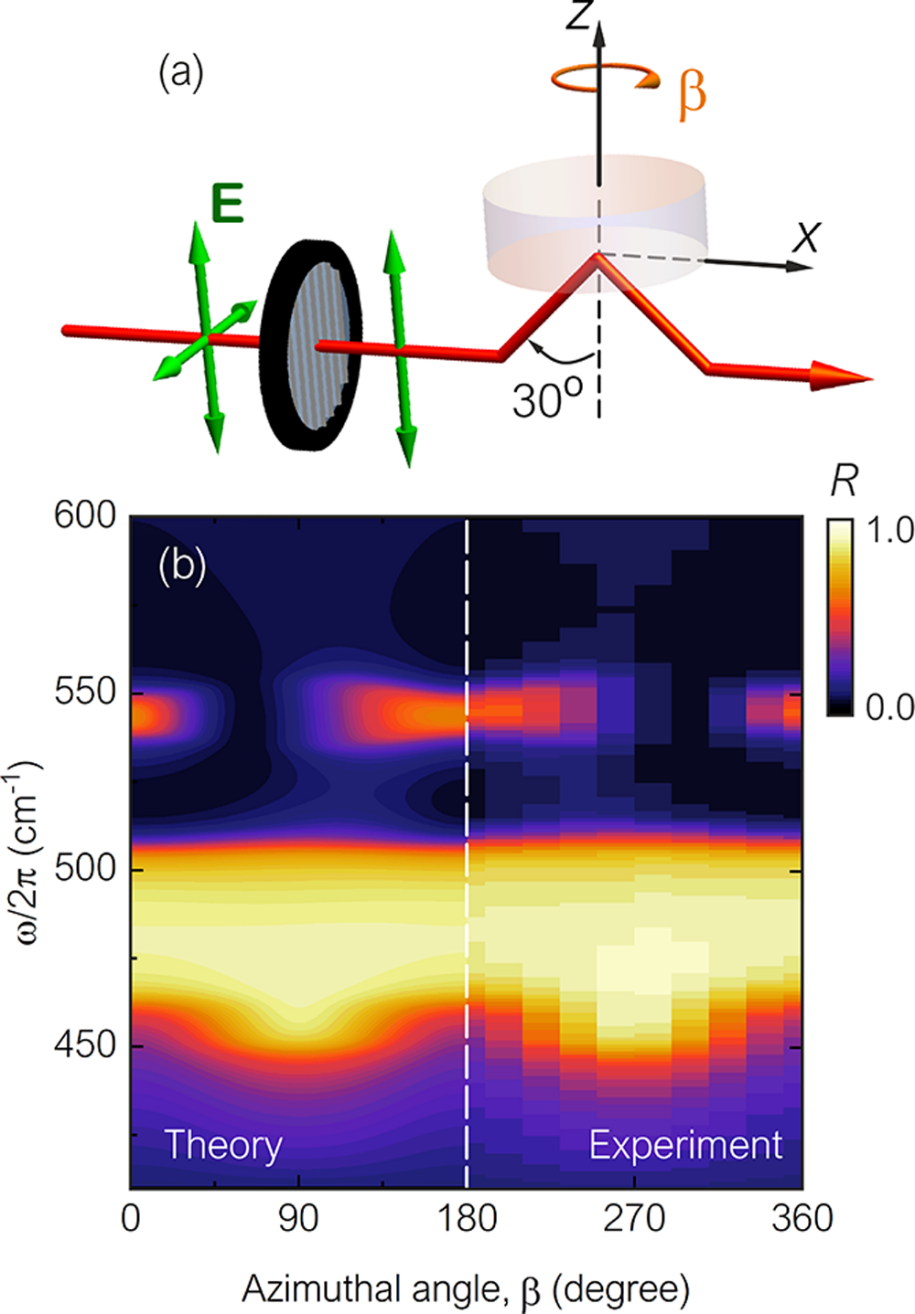
(a) Experimental setup geometry, where a TM-polarized beam is incident at the surface of crystal quartz with θ = 30°. (b) Theoretical and experimental reflectance spectra for an anisotropy rotation angle of φ = 45°.

To confirm the asymmetric nature of the reflection in bulk hyperbolic materials, we have performed infrared reflection measurements where the beam is incident at a fixed angle of θ = 30° and the incident wave is TM-polarized (as shown in the schematics in Figure 3a, where a polarizer is placed in the beam path between the sample and the unpolarized infrared radiation source). The spectra of reflectance for a 360° rotation of the incidence plane, β, is given in Figure 3b for the bending angle φ = 45°. We can see that if we reverse the direction of propagation for β = ±45° (or 45° and 225° in the plot) the reflectance will not be the same. In fact, for β = +45° it will be at a minimum around 540 cm^–1^, while there will be a strong response for β = −45°, which is in excellent agreement with the data from calculations provided in Figure 2b,c. Note that the full experimental spectrum (for a full 360° rotation) is provided in the Supporting Information.

### Physical Mechanism for Asymmetric Reflection

The Helmholtz reciprocity principle dictates that the position of the source and detector in a particular optical setup can be exchanged without altering the observed intensity. Still, we have just seen that asymmetric reflection for opposite incident angles can be introduced to a reciprocal hyperbolic crystal, which then begs the question “what is the mechanism responsible for such behavior?”. So, let us address how the strong asymmetric reflection found in this work is not only possible, but also how it does not violate the Helmholtz reciprocity principle.

**Figure 4:**
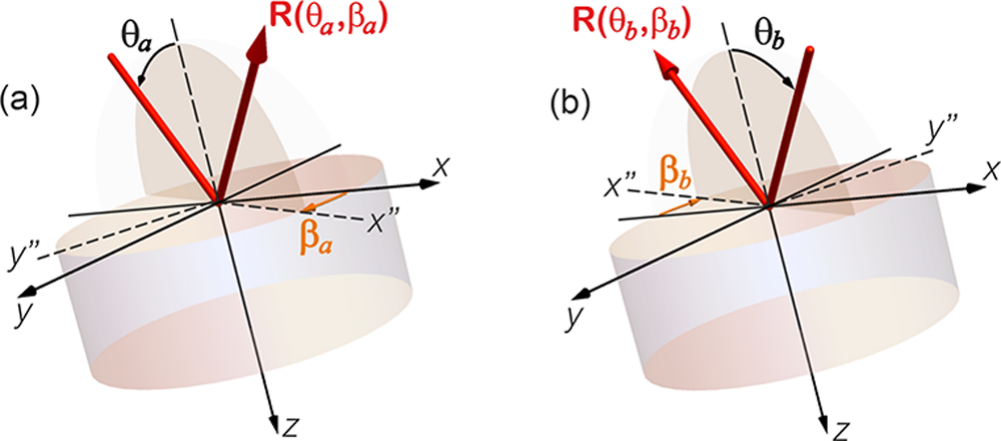
Geometry of reflection for two special cases of the incident angle, θ, and rotation of the incidence plane, i.e., azimuthal angle β, we termed (a) *R*(θ_*a*_, β_*a*_) and (b) *R*(θ_*b*_, β_*b*_).

We start by taking the geometries of reflection shown in Figure 4, which are special cases of a linearly polarized infrared beam incident at the surface of a hyperbolic material where the plane of incidence is allowed to rotate, as discussed earlier. Mathematically, for an opaque, anisotropic material with a smooth interface, the reflectance *R* for a TM-polarized beam at incident angle θ_*a*_ and azimuthal angle β_*a*_, as shown in Figure 4a, can be written in its most general form as^33^
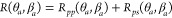
1where *R*_*pp*_(θ_*a*_, β_*a*_) = |*r*_*pp*_(θ_*a*_, β_*a*_)|^2^ and *R*_*ps*_(θ_*a*_, β_*a*_) = |*r*_*ps*_(θ_*a*_, β_*a*_)|^2^. Here, *r*_*pp*_ and *r*_*ps*_ are the TM and TE components of the complex amplitude reflectivity coefficients which can be calculated using standard transfer matrix theory as described in Materials and Methods.

Similarly, at incident angle θ_*b*_ and azimuthal angle β_*b*_, as shown in Figure 4b, the reflection for a TM-polarized beam can be written as
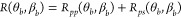
2where *R*_*pp*_(θ_*b*_, β_*b*_) = |*r*_*pp*_(θ_*b*_, β_*b*_)|^2^ and *R*_*ps*_(θ_*b*_, β_*b*_) = |*r*_*ps*_(θ_*b*_, β_*b*_)|^2^. Here, (θ_*a*_, β_*a*_) and (θ_*b*_, β_*b*_) are a pair of specular incidence, and reflection, angles. Hence, θ_*a*_ = θ_*b*_, and β_*b*_ = β_*a*_ + 180°.

So, let us pause here and briefly investigate reciprocity. In order to satisfy the Helmholtz reciprocity principle, it is necessary to have^28^
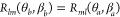
3that is
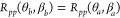
4and
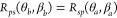
5

Thus, if we subtract the reflectance coefficients *R*(θ_*a*_, β_*a*_) and *R*(θ_*b*_, β_*b*_) from each other, we obtain
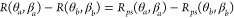
6

Now, from this, there are two main conclusions to be drawn:

(1) The coefficients *R*_*ps*_(θ_*a*_, β_*a*_) and *R*_*ps*_(θ_*b*_, β_*b*_) are indeed allowed to be different from one another without breaking the Helmholtz reciprocity principle.
(2) If they do differ from one another, the total reflectance coefficients *R*(θ_*a*_, β_*a*_) and *R*(θ_*b*_, β_*b*_), sketched in Figure 4, will also be different from each other.

Typically, from a simple material, there is no TE component for the reflection of a TM polarized incident wave, or vice versa, and thus, eq 6 = 0. Therefore, from this, we must then infer that the effect we have just shown in our initial experiment is a consequence of cross-polarization conversion between TM waves and transverse electric (TE) waves, which only arises at certain azimuthal angles β and that is mediated by the bending angle φ. In the initial experiment we performed, we considered the incident wave to be TM-polarized. Therefore, the reflected wave must contain TM and TE components due to such cross-polarization conversion effects allowed by eq 6 in order for its asymmetry to take place but still satisfy the reciprocity principle. This asymmetry without breaking the reciprocity principle is, in fact, somewhat analogous to one of the cases of asymmetric transmission discussed by Carloz and co-workers,^51^ where the system is reciprocal, but optical components in the beam path are designed in such a geometry that enables direction-dependent light propagation. In our case, there is no such optical components. The phenomenon we discuss is perhaps closer to that wherein polarization conversion was used to achieve asymmetric transmission in large-area meta-surfaces.^52^ However, here the interplay between the anisotropy direction and the rotation of the incidence plane (φ and β, respectively) are the enablers for such effects through polarization conversion of a linearly polarized incident beam. We should note that, by definition, for this reciprocal system, the TE-polarized reflection of TM-polarized incident light at an angle θ must be equal to the TM-polarized reflection of TE-polarized incident light at the same angle θ.

More generally, the implications of eq 6 can be summarized as follows:

- First, when the anisotropy is perpendicular to the crystal’s surface (φ = 0), TE and TM waves are decoupled, and there is no cross-polarization conversion between them; thus, *R*_*ps*_(θ_*b*_, β_*b*_) and *R*_*ps*_(θ_*a*_, β_*a*_) are both zero.^53^ Hence, the reflection is independent of the azimuthal angle β (as we earlier inferred based purely on the geometry of crystal quartz).
- Second, when the anisotropy is parallel to the crystal surface, φ = 90°, *R*_*ps*_(θ_*b*_, β_*b*_) = *R*_*sp*_(θ_*a*_, β_*a*_).^53^ This means that while the incident beam might be TM-polarized, there is polarization conversion in the reflected beam. However, if we use this condition and by substituting eq 5 into eq 6 we arrive at *R*(θ_*a*_, β_*a*_) = *R*(θ_*b*_, β_*b*_). This means that the reflection is then symmetric in the *k*-space, albeit mixed polarized.
- And finally, when the anisotropy is at an arbitrary angle ϕ ≠ 0, together with a nonzero azimuthal angle β, *R*_*ps*_(θ_*b*_, β_*b*_) does not equal *R*_*ps*_(θ_*a*_, β_*a*_) and, hence, the reflection is asymmetric in the wavevector space.

**Figure 5:**
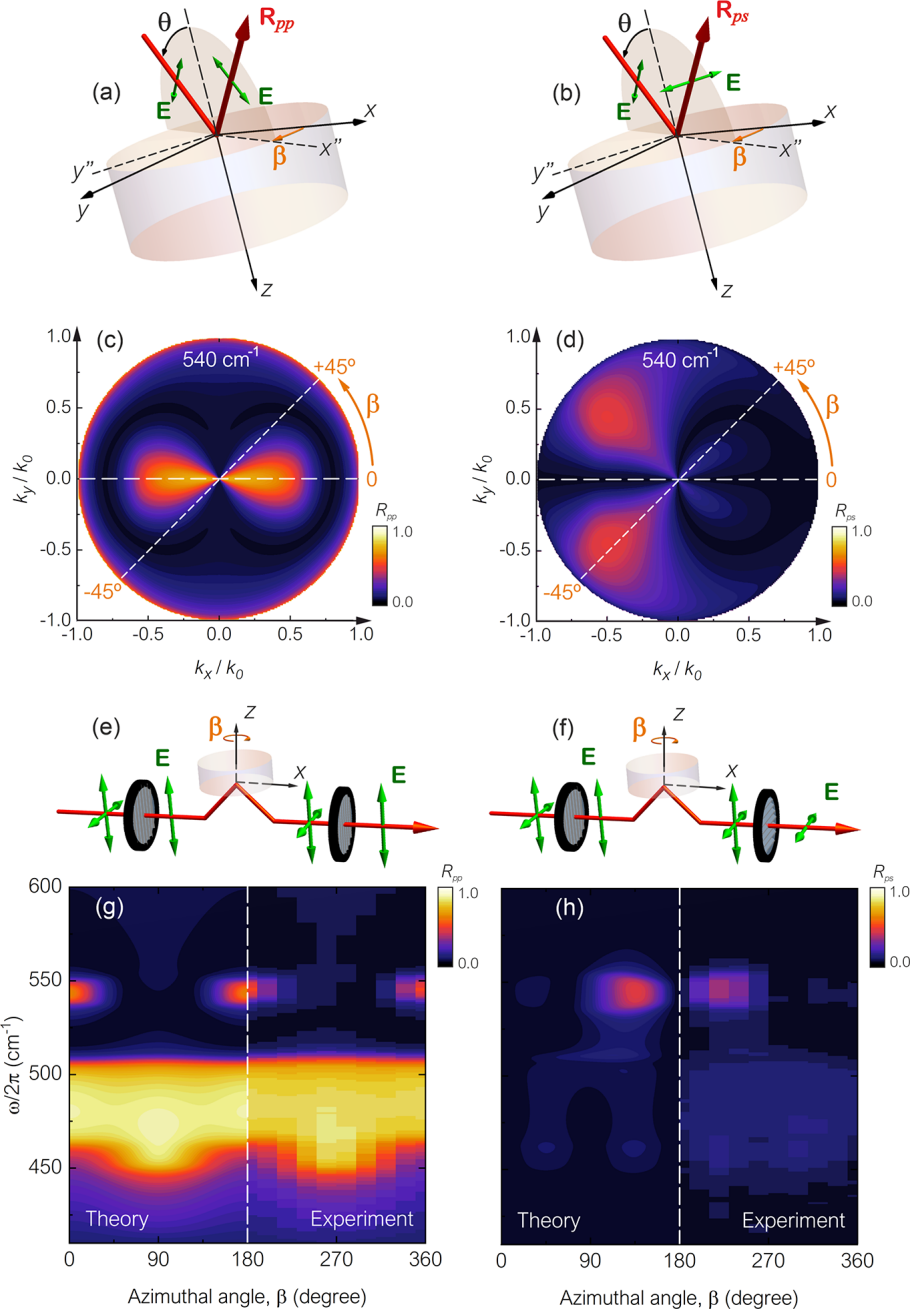
Reflection geometry for (a) TM polarization and (b) TE polarization selections. (c) TM and (d) TE polarized reflection spectra as a function of the wavevector in the *k*_*x*_–*k*_*y*_ space at ω/2π = 540 cm^–1^. Experimental set up for a TM-polarized wave incident at θ = 30° and with output (e) also TM-polarized and (f) TE-polarized. (g) Experimental and theoretical results for the TM and (h) TE components of the reflection. All data are for a bending angle of φ = 45°.

In order to further test the conclusions obtained from the analytical description above, we separate the TM (*R*_*pp*_) and TE (*R*_*ps*_) components of the reflected waves, as shown in Figure 5a and b, respectively. For a direct comparison, we use the same example as shown in Figure 2c of the reflectance in the *k*-space for ω/2π = 540 cm^–1^. One can see that the TM component (shown in Figure 5c) satisfies eq 4, that is, *R*_*pp*_ is symmetric. The TE component (*R*_*ps*_) on the other hand is highly asymmetric on *k*_*x*_, as shown in Figure 5d. In particular, we use the short dashed line to highlight again that for β = +45° there is no strong response, while for its negative counterpart, β = −45°, a strong signal is observed for *R*_*ps*_. Hence, the total reflectance *R* is highly asymmetric on *k*_*x*_, as shown in Figure 2b,c and confirmed experimentally in Figure 3b.

We now have even stronger theoretical evidence that the asymmetry indeed comes from the cross-polarization conversion, where our numerical simulations agree well with our analytical analysis. To test this, we performed further infrared reflection measurements, but this time, the TM and TE components of the reflected wave were then separated with a second polarizer as shown in the setup schematics. Two experiments were performed where both start with a polarizer placed in the beam path to generate TM incident radiation at the sample surface. We then placed a second polarizer in the beam path between the reflected beam and the detector; one polarizer selected TM reflection (*R*_*pp*_, as shown in Figure 5e) and the other selected TE reflection (*R*_*ps*_, as shown in Figure 5f). The results for each case are shown in Figure 5g,h for TM- and TE-polarized reflections, respectively. Much like Figure 3, the left-hand side shows the theoretical results, and the right-hand side shows the experimental data (see Supporting Information for full experimental spectra).

In both cases, the experiment results agree remarkably well with the simulation results in that the TE component is asymmetric, while the TM component is symmetric; as a result, the total reflection is asymmetric. The experimental results thus confirm that the asymmetry stems from the polarization conversion. We find it worth noting that hyperbolic materials are typically characterized by being either Type I or Type II, the former being those possessing ε_∥_ < 0 and ε_⊥_ > 0 and the latter have ε_∥_ > 0 and ε_⊥_ < 0.^49^ Here, the strongest asymmetric features in the reflectance are mostly observed within the region where the crystal behaves as a Type II hyperbolic material. While it would be natural to speculate that the asymmetry observed here would require Type II behavior, this is not required by the polarization conversion argument detailed from eqs 1–6. However, the asymmetry in the hyperbolic dispersion, from positive to negative *k*_*x*_, has been shown to be directly responsible for asymmetric transmission^34^ and absorption^35^ in systems where no rotation of the incidence plane takes place. Thus, the behavior of the hyperbolic dispersion should also affect the asymmetric reflection and its extent deserves further investigation.

## Discussion and Conclusions

**Figure 6:**
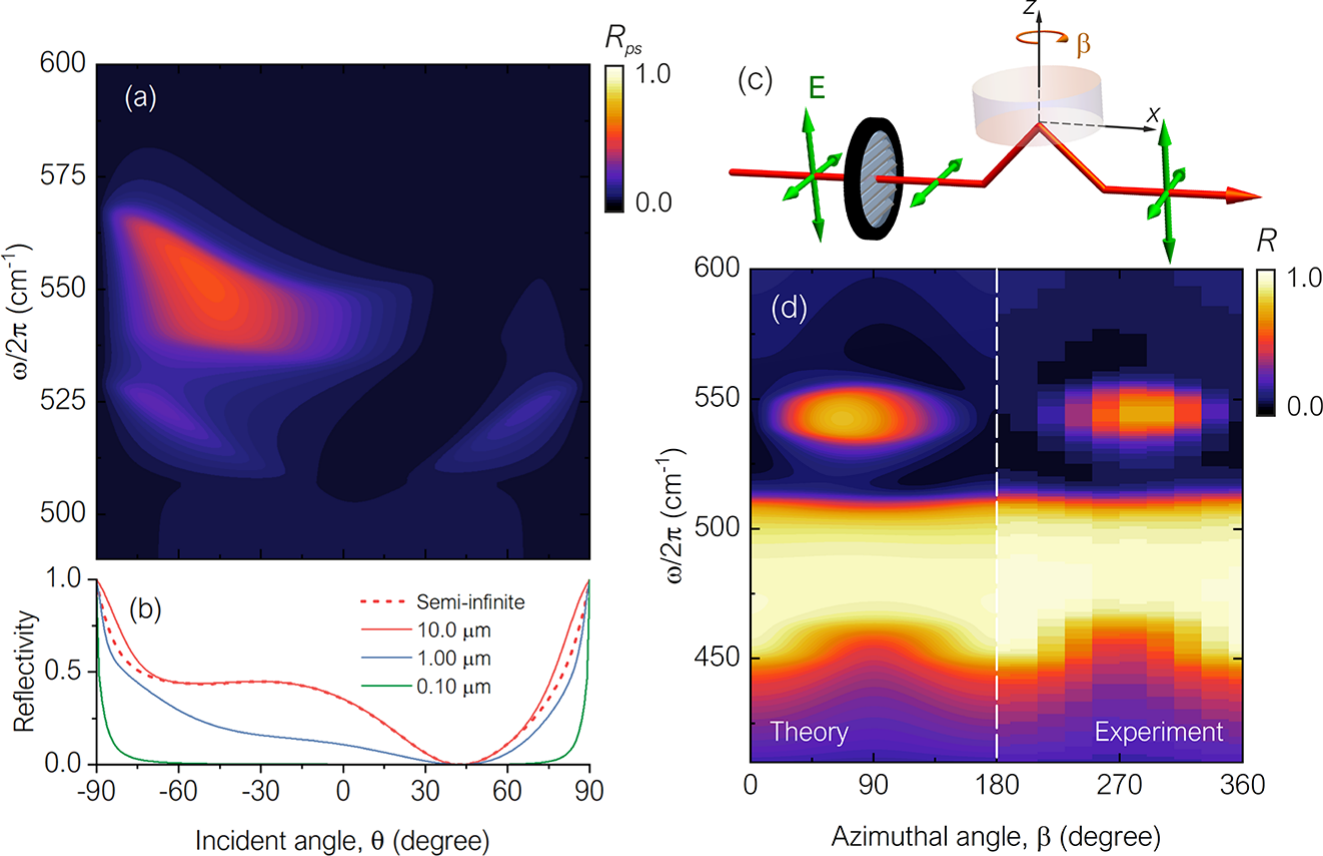
(a) TE component in the reflected wave of the semi-infinite crystal quartz as a function of the incident angle and the wavenumber. (b) The reflection as a function of the incident angle for different thicknesses of crystal quartz. (c) Schematics for reflection measurement with a TE-polarized incident beam. (d) The reflection from crystal quartz using the setup in (c) as a function of the azimuthal angle β.

Here we have demonstrated that strong asymmetric reflection can be achieved in reciprocal hyperbolic materials, such as crystal quartz, due to cross-polarization conversion. This strong cross-polarization conversion takes place near the epsilon-near-zero point, which has been observed in other epsilon-near-zero materials before and exploited for a variety of applications.^54−56^ We note that in our case, if we take only the TE-polarized reflection component of Figure 2b, there will be a strong region where reflection can be fully blocked for positive incident angles, but not so for their negative counterpart, as shown in Figure 6a. This further highlights how the mechanism we used here can be employed to engineer efficient “one-way” optical devices.

For the entirety of this work, we have concentrated on the behavior of a semi-infinite material. This allowed us to achieve the unusual state of bulk asymmetric reflection. However, the thickness of a crystal or film can also be used to control the asymmetry. For instance, in Figure 6b we show the reflectance as a function of the incident angle for different thicknesses of crystal quartz at wavenumber 540 cm^–1^. One can see that the asymmetry becomes stronger when the thickness increases from 1 to 10 μm, but at this particular frequency it does not change substantially when the thickness is larger than 10 μm. Note that while the thickness-dependent behavior may vary with frequency, we have used a crystal that is hundreds of times larger than the wavelengths taken here, therefore being well within the semi-infinite limit. It is worth mentioning that when considering finite materials, other asymmetric light propagation effects, such as asymmetric absorption, may also be introduced through the combination of plane of incidence rotation and anisotropy rotation. This has so far been investigated for finite systems where the anisotropy is rotated, but no effects of rotating the plane of incidence have been considered.^35^ A combination of these two might lead to a new degree of controllability of wave propagation in finite hyperbolic crystals.

We should also note that while we have focused our discussion around TM-polarized incident waves, the same behavior should take place for TE-polarized waves (see schematics in Figure 6c). As an example, in Figure 6d we show the reflectance for a TE-polarized incident beam with its output containing both TE and TM components. In this case, we can indeed see that there should be an asymmetry at, for instance, ±45°. As discussed in eqs 1–6, the origin of this is also the conversion of polarization once the azimuthal and the bending angles are nonzero.

Finally, compared with magneto-optical materials, reciprocal hyperbolic crystals such as quartz can achieve asymmetric absorption, transmission, and reflection without applied magnetic fields. Thus, this class of materials could potentially serve as a suitable alternative to magneto-optical materials at far-infrared frequencies with more versatility. By not needing external fields to operate, this class of hyperbolic media could be even more easily incorporated into electronic devices. The use of an azimuthal angle to achieve said functionality is somewhat reminiscent of the “magic angle” recently employed in two-dimensional systems in order to obtain new-wave propagation.^57^ Therefore, our findings should also be of interest for the new physics and technology currently under development using van der Waals structures. We believe that such simple uniaxial crystals may also find important applications in unidirectional emission and unidirectional and transformation optics.

## Materials and Methods

### Theory

To calculate the reflectivity coefficient *r*, and subsequently the reflectance *R*, we employed the standard 4 × 4 transfer matrix method. This method is a relatively straightforward technique for obtaining the exact solution of Maxwell’s equations for the electromagnetic propagating in multilayer structures, which has been detailed elsewhere.^32,58−60^ In each layer, the electric and magnetic fields can be expressed as a sum of waves propagating upward and downward. By matching the boundary condition for the fields propagating up and down at each boundary (the tangential components of the magnetic and electric field vectors which should be continuous at the interface), the reflection can be obtained. We should note that in our geometry, due to the rotation of both plane of incidence and anisotropy with respect to the crystal surface, the coupling of both TE and TM waves with the rotated permittivity tensor needs to be taken into consideration. The transfer matrix method has been shown to be robust enough to handle said situations.^61^

All parameters and equations for the dielectric tensor components of the crystal quartz are readily available in the work of Estevam and colleagues.^42^ Note that, for a better fit with the experimental results, we have also used the parameters fitted by Estevam et al.^42^ based on those originally obtained by Gervais and Piriou;^62^ a table comparison of both sets of parameters is collated in ref (42). It is also noteworthy that while the dielectric tensor components shown in Figure 1a appear to be single-poled Lorentz curves, we have used all appropriate infrared-active phonon modes for the calculations given in the references above; those just do not appear in the frequency range of interest here, as they span all the way throughout far- to mid-infrared regions.

### Experiment

All experimental data presented was obtained using Fourier-transform infrared spectroscopy measurements of reflectance, *R* = rr*, using a Bruker Vertex 70 spectrometer. The spectra have a resolution of 2 cm^–1^ and each spectrum was averaged 16 times. We used a KRS-5 polarizer placed in the path of the incident to obtain either TM- or TE-polarized radiation, as shown in Figure 3a. For the data shown in Figure 5, another KRS-5 polarizer was placed in the path of the reflected beam to obtain either TE- or TM-polarized outputs only, as shown in Figure 5g,f. The samples used were chemically polished crystal quartz flat slabs of 20 mm diameter and thickness *d* = 1 cm (as shown in Figure 6b, this thickness is sufficient to achieve semi-infinite behavior), obtained from Boston Piezo Optics Inc. For the anisotropy rotation at 45°, the orientation of the anisotropy axis was chosen with respect to the crystal’s surface, being precisely determined by single crystal X-ray diffraction measurements with the crystal mounted on a goniometer, which allowed the positioning of the crystal at selected orientations. The azimuthal angle rotation was performed manually by rotating the crystal quartz in plane every 20° (while we believe this to be fairly accurate, an error of ±2.0° is possible but should not make too much of a difference when plotting the reflectivity as intensity maps such as done above).

## Supporting Information Available

The full (360 rotation) version of all experimental plots shown in the main article (PDF)
