# Supplementary material for: Asymmetric Reflection Induced in Reciprocal Hyperbolic Materials
Source: ACS Photonics. 2022 Jul 20;9(8):2774–82. doi: 10.1021/acsphotonics.2c00551 (PMC9389604; doi:10.1021/acsphotonics.2c00551)
Supplement: Supplementary file 1 — ph2c00551_si_001.pdf [file ph2c00551_si_001.pdf]

Supplementary Information for

Asymmetric Reflection Induced in Reciprocal Hyperbolic Materials

Xiaohu Wu,[†] Cameron A. McEleney,[‡] Zhangxing Shi,[†] Mario Gonz´alez-Jimenez,[¶] and Rair Macêdo[*,‡]

[†]*Shandong Institute of Advanced Technology, Jinan 250100, Shandong, China*

[‡]*James Watt School of Engineering, Electronics & Nanoscale Engineering Division, University of Glasgow, Glasgow G12 8QQ, United Kingdom*

[¶]*School of Chemistry, University of Glasgow, G12 8QQ, United Kingdom*

E-mail: Rair.Macedo@glasgow.ac.uk

This file contains two pages and 2 figures: S1 (one panel) and S2 (two panels) with expanded experimental data for the results shown in the main article.

As seen in the main text, the experimental and theoretical spectra are rather similar. Thus, to avoid repetition, we have showed the experimental and theoretical data as two halves of the same plot. For completeness, here we give the full experimental spectra (360º rotation of the incidence angle) for all figures shown in the main text.

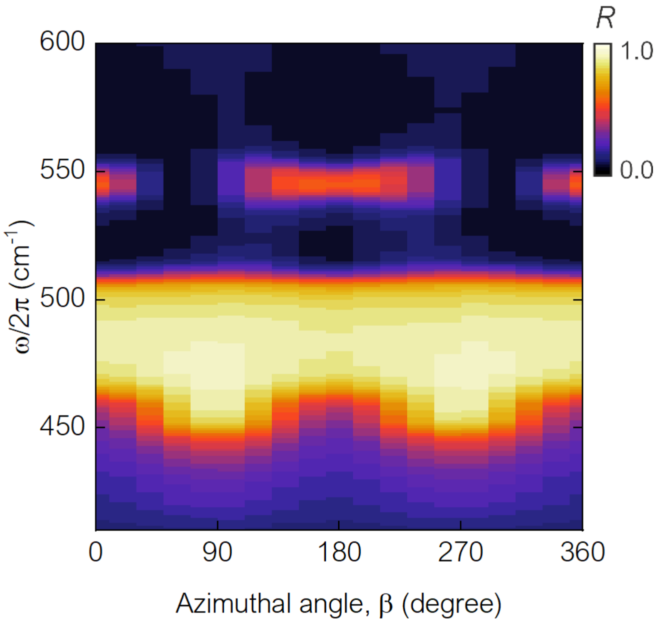

Figure S1: Experimental reflectance spectra for a TM-polarized wave is incident at the surface of crystal quarts with $\theta = 30^o$ for the anisotropy rotated by of $\varphi = 45^o$. This is equivalent to that shown in Fig. 3(b).

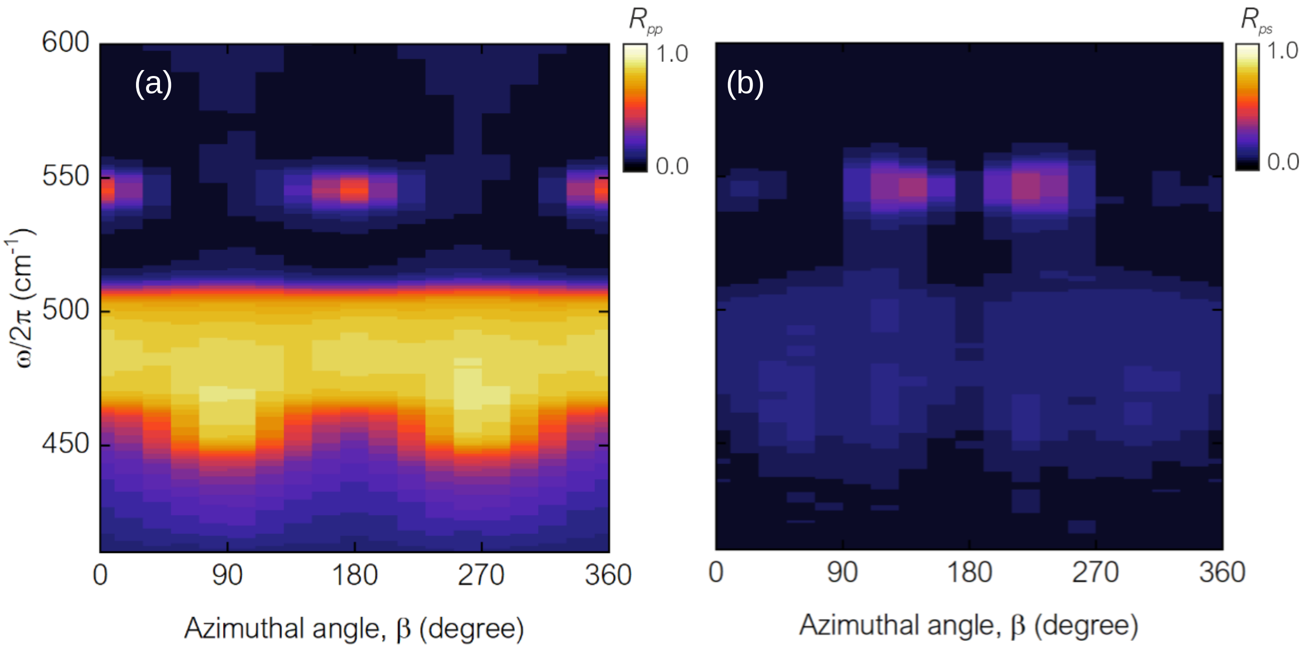

Figure S2: Experimental reflectance spectra for a TM-polarized wave is incident at the surface of crystal quarts with $\theta = 30^o$ for anisotropy rotate by an angle $\varphi = 45^o$ and its (a) TM-polarized and (b)TE-polarized output. Equivalent to Figs. 5(g) and (h), respectively.
